# A physiological time analysis of the duration of the gonotrophic cycle of *Anopheles pseudopunctipennis *and its implications for malaria transmission in Bolivia

**DOI:** 10.1186/1475-2875-7-141

**Published:** 2008-07-26

**Authors:** Frédéric J Lardeux, Rosenka H Tejerina, Vicente Quispe, Tamara K Chavez

**Affiliations:** 1 Institut de Recherche pour le Développement (IRD), C.P. 9214, La Paz, Bolivia and BP 64501, 34394, Montpellier, Cedex 5, France; 2 Laboratorio de Entomología Medica, Instituto Nacional de Laboratorios de Salud (INLASA), Casilla M-11, Miraflores, La Paz, Bolivia

## Abstract

**Background:**

The length of the gonotrophic cycle varies the vectorial capacity of a mosquito vector and therefore its exact estimation is important in epidemiological modelling. Because the gonotrophic cycle length depends on temperature, its estimation can be satisfactorily computed by means of physiological time analysis.

**Methods:**

A model of physiological time was developed and calibrated for *Anopheles pseudopunctipennis*, one of the main malaria vectors in South America, using data from laboratory temperature controlled experiments. The model was validated under varying temperatures and could predict the time elapsed from blood engorgement to oviposition according to the temperature.

**Results:**

In laboratory experiments, a batch of *An. pseudopunctipennis *fed at the same time may lay eggs during several consecutive nights (2–3 at high temperature and > 10 at low temperature). The model took into account such pattern and was used to predict the range of the gonotrophic cycle duration of *An. pseudopunctipennis *in four characteristic sites of Bolivia. It showed that the predicted cycle duration for *An. pseudopunctipennis *exhibited a seasonal pattern, with higher variances where climatic conditions were less stable. Predicted mean values of the (minimum) duration ranged from 3.3 days up to > 10 days, depending on the season and the geographical location. The analysis of ovaries development stages of field collected biting mosquitoes indicated that the phase 1 of Beklemishev might be of significant duration for *An. pseudopunctipennis*. The gonotrophic cycle length of *An. pseudopunctipennis *correlates with malaria transmission patterns observed in Bolivia which depend on locations and seasons.

**Conclusion:**

A new presentation of cycle length results taking into account the number of ovipositing nights and the proportion of mosquitoes laying eggs is suggested. The present approach using physiological time analysis might serve as an outline to other similar studies and allows the inclusion of temperature effects on the gonotrophic cycle in transmission models. However, to better explore the effects of temperature on malaria transmission, the others parameters of the vectorial capacity should be included in the analysis and modelled accordingly.

## Background

In mosquito females, the blood meal is essentially used to provide energy for the maturation of eggs. Therefore, digestion and ovaries development are physiologically integrated. Many females blood sucking insects will develop and lay a batch of eggs each time a sufficient blood meal is taken. This is called the gonotrophic concordance [1]. Throughout the mosquito's life, the gonotrophic cycle will be repeated and consisted in three successive biological phases (Beklemishev phases) [2], which are: (i) the search for a host and blood-feeding, (ii) digestion of the blood and egg maturation, and (iii) the search for a suitable oviposition site and oviposition. The gonotrophic cycle duration may then be defined as the time interval between two consecutive blood-meals (or the time interval between two consecutive acts of egg-laying). In this cyclic mode of reproduction the biting frequency is determined by the length of the gonotrophic cycle (*i.e*. one bite with a complete blood meal per cycle). The measurement of the biting frequency is an important parameter in transmission models and in formulas such as the vectorial capacity [3], which predict the vector's pathogen-transmitting potential. Indeed, the vectorial capacity *C*, which may be defined as the daily rate at which future inoculations arise from a currently infective human case, varies according to the square of the human-biting habits of the vector, *a*. For malaria transmission, it may have the following formulation:

(1) *C *= *m*.*a*^2^.*p*^*x*^/(-*Log*(*p*))

where

*a = *Number of blood-meals taken on humans by a mosquito per 24 hours

*m = *The relative vector density (*i.e*., the number of vector per man)

*p = *daily survival rate of the mosquito

*x = *duration of the extrinsic cycle of the transmitted parasite (*e.x. Plasmodium sp*. in case of malaria)

In fact, the human-biting habits of the vector (*a*) depends on the frequency of biting, *i.e*., on the duration (*g*) of the gonotrophic cycle:

(2) *a = HBI/g*

where

*HBI *= Human Blood Index, *i.e*. the proportion of blood meals taken on humans in a population of mosquitoes

*g *= duration of the gonotrophic cycle (in days)

As such, *C *may be rewritten as:

(3) *C *= *m*.(*HBI*/*g*)^2^.*p*^*x*^/(-*Log*(*p*)) = *φ*/*g*^2^

where

*φ *captures all others parameters except *g*

From (3) it can be seen that when the duration of the gonotrophic cycle *g *is doubled (for example, increasing from two to four days), the vectorial capacity *C *is divided by four! This simple deterministic analysis indicates that *g *may have impacts on the vectorial capacity and on the transmission dynamics. Indeed, because most of the parameters may vary accordingly to external factors (such as temperature) there can be some opposite (or on the contrary synergistic) impacts on the vectorial capacity. Caution should then be taken when only one parameter of *C *is analysed alone.

The gonotrophic cycle duration may also be used to compute estimates of the daily survival rate *P *of mosquitoes [4] using:

*P *= *Q*^1/*g*^

where

*Q *= proportion of parous mosquitoes females in the population

*g *= duration of the gonotrophic cycle (in days)

Once again, if g is not computed with enough precision, daily survival rate estimates may be inconsistent.

Therefore, precise computation of *g *as well as a correct analysis of its variations with time and its co-variations with other parameters are essential in pathogen transmission studies and modelling. To estimate *g*, various techniques are available. The most direct and usual techniques that take into account the three biological phases of Beklemishev and all the environmental factors which can influence the estimation of *g*, are field mark-recapture techniques [5,6], sometimes associated with mathematical models [7-9]. However, reliable estimates need substantial capture rates, and when recaptures are scarce, other estimating techniques have been proposed, including the measurement of correlated variables such as the extrinsic cycle duration of a parasite [10], or variations in the proportion of parous females with time and regression modelling [11-13]. Nevertheless, reliable field data may be difficult to obtain and frequently, the gonotrophic cycle duration is estimated through laboratory experiments, ignoring ecological influencing factors and mosquito behaviour that induce delays between oviposition and refeeding, or time to locate a suitable site for oviposition [14]. In this case, the gonotrophic cycle duration is simply equated to the time for blood-engorged females to become fully gravid (phase 2 of Beklemishev), which can under-estimate its true value.

The phase 2 of Beklemishev depends entirely on physiological processes and as such is temperature dependant in mosquitoes which are poikilothermous organisms. Temperature is a key factor in arthropod development and accounts for most variation in the gonotrophic cycle duration [15]. The easiest way to correlate temperature and development (or any development phase) is the use of the concept of day-degrees, assuming that the development rate is a linear function of temperature. This may be (approximately) the case in some part of the temperature-rate curve where temperatures are moderate. Then, one day-degree is the amount of development that occurs in one day (24 hours) when the temperature is one degree above the development threshold. The method has been used extensively in many entomological domains (especially in agriculture pest warnings), using several refinements for computation [16]. However, over the full range of physiologically viable temperatures, the rate of development generally follows a S-hook shaped function of increasing temperatures, with no or very low development at low temperatures, an accelerated development above a minimum temperature threshold up to a maximum, then a retarded development at very high temperatures, and a sharp drop near the upper limit of survival [17,18]. Therefore, because of the non-linearity of the relationship between developmental rates and temperatures, the accumulation of day- degrees is erroneous. The concept of physiological time (*i. e*., the amount of time needed to complete development) can overcome this problem. As compared to the day-degree concept, it has the following advantages: (i) it makes no arbitrarily assumptions on the shape of development rate function, and (ii) it admits the effect of other factors besides temperature on the rate of development. Several models have been proposed to describe development rates as a function of temperature and have been successfully used in entomology to compute physiological time [19-21].

The present study claims to present an analysis of the duration of the gonotrophic cycle of *An. pseudopunctipennis *according to the temperature, using the concept of physiological time. Laboratory experiments of mosquito egg development were used under different constant temperatures to derive parameters for a mathematical model. The model, validated for use with variable temperatures as observed in the field, predicts the length of the gonotrophic cycle (mainly Beklemishev's phase 2 which encompass the physiologically temperature dependant processes). If the entire gontorophic cycle is to be estimated, Beklemishev's phases 1 and 3 should be incorporated. Therefore, to explore potential bias in the model predictions due to not taken into account these phases, their qualitative analysis is carried out using data on ovaries development from biting *An. pseudopunctipennis *captured in the field. The model is then used with field temperature records in four characteristic sites of Bolivia (malaria endemic and non-endemic) to predict the range of the gonotrophic cycle length and its variations within seasons. Model predictions are discussed in relation to possible co-variations with other biological parameters of the vectorial capacity.

## Methods

### Modelling time of egg maturation

#### The physiological time concept and modelling of development time

The rate of development can be measured as the reciprocal of the number of time units that is required for completion of development. It can be estimated for the entire ontogenesis or for a specific stage, as for example, the time of maturation of eggs in mosquitoes (in laboratory experiments, this time of maturation is equated to the time from blood engorgement to egg-laying). The progress in physiological development can be measured on a physiological time basis which is not linear as calendar time, but is faster when temperatures are high and slower when it is cold. Therefore, the development rate can be defined as the fraction of development period that occurs in one calendar day. For example, if the rate of development is 0.05 per day and by definition, development is achieved at 100%, then in one day the insect increments its physiological time by 5%.

In order to determine the duration of development, development rates can be accumulated with calendar time in a way similar to the degree-day concept. Entire development is reached when the accumulated rates are 1 (*i.e*., 100%) and the mathematical expression is:

(4) $$\int_{0}^{\alpha }r(T(t)).dt=1$$

where

*r*(*T*(*t*)) is the development rate as a function of temperature *T *which itself is dependent on time *t*,

*α *is the complete development time, the parameter one wants to estimate.

To facilitate computation, the above equation can be rewritten for discrete time intervals as:

(5) $$\sum_{0}^{\alpha }r(T(t)).\Delta t≅1$$

where

Δ*t *are periods of constant temperature (in field conditions, the Δ*t *are chosen as periods small enough to consider that temperature is constant in that time interval, for example one hour)

At each time *t *of the day there is a corresponding temperature *T(t) *and a corresponding rate of development *r(T(t))*. So, to compute *α*, one has to solve iteratively equation (5) by summing small amounts of development for small periods of time where temperature is considered constant, and stopping the summations at step *α*, when the total is 1.

The model needs the function *r(T)*, *i.e*., the development rate *r *at temperature *T*. Several functions have been suggested [19], but that of Lactin *et al *[20], a modification of Logan *et al *function [22] has proved to adequately describe various situations and was then chosen in the present study.

This function expresses *r(T) *as:

(6) $$r(T)=\exp(\rho T)-\exp\left[\rho Tm-\frac{\left(Tm-T\right)}{\Delta }\right]+\lambda$$

where

*T*_*m *_is a thermal maximum, *i.e.* the "lethal" temperature at which life processes cannot be sustained for prolonged periods of time,

Δ is the temperature range over which "thermal breakdown" becomes the overriding influence,

*ρ *is a parameter that can be interpreted as a composite value for critical enzyme-catalysed biochemical reactions,

*λ *is the value of *r(T*_*m*) _(i.e. when *T = T*_*m*_) and allows the curve to intersect the abscissa at sub-optimal temperatures, permitting the estimation of the base temperature (*T*_*base*_) (*i.e*., the temperature below which development stops) by allowing *r(T) *= 0 to be solved numerically for temperature.

**Figure 1:**
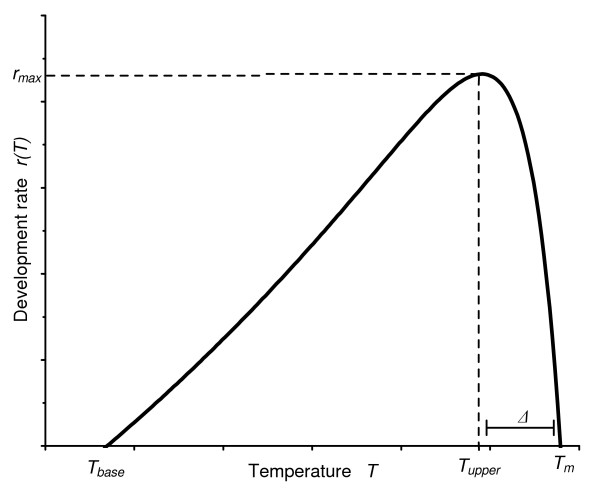
**A generalized insect developmental rate curve as a function of temperature: the Lactin et al. function **[20]. Descriptive parameters are *T*_*base*_, the base temperature below which development does not proceed, the maximum development rate *r*_*max *_and its corresponding temperature *T*_*upper*_, the width *Δ *of decline phase in developmental rate above optimum temperature and the thermal maximum *T*_*m*_.

The graphical representation of such a function is given in Figure 1. To estimate the four parameters of the function for *An. pseudopunctipennis*, insects were reared at a series of constant temperatures *T*_*i *_and the rate of development *r*_*i *_at each temperature *T*_*i *_was recorded (see details in next section). The function *r(T) *is non-linear in its parameters, but with the series of observed points (*r*_*i*_, *T*_*i*_), the parameters *T*_*m*_, Δ, *ρ *and *λ *were estimated using the Simulated Annealing method [23] implemented in the GOSA software (Bio-Log scientific software, France).

The function also permits the computation of the upper threshold, *T*_*upper*_, which is the temperature value for which the development rate is maximum. In a mathematical sense, the first derivative of *r(T) *is equated to zero and solved for *T*. The value is then:

(7) $$T_{upper}=\frac{\Delta .Ln(\Delta .\rho )}{\left(1-\Delta .\rho \right)}+T_{m}$$

To sum up, the general model of physiological time for gonotrophic cycle development is constituted by equation (5), which has to be solved iteratively to estimate the cycle duration. Equation (5) is based on equation (6), which gives the development rate of an insect as a function of temperature. Therefore, parameters values of equation (6) have first to be estimated for each species under study. This has been done for *An. pseudopunctipennis *(see next section).

#### Parameterizing the model: Laboratory observation of egg maturation at different temperatures

To obtain the duration of egg maturation at constant temperatures and therefore to parameterize equation (6) for *An. pseudopunctipennis*, several experiments were carried out as follow. For each experiment, 70 to 200 *females *from the Mataral strain (from Mataral village, Bolivia, and reared in the insectary since 2003) were force-mated [24] and blood-fed on rabbits. They were immediately kept in climatic chambers (Meditest 600/1300, Firlabo, France, or Binder KBWF 720, Tuttlingen, Germany) in individual oviposition vials of ≈100 ml with cotton soaked with water and covered with filter paper to facilitate the mosquito egg-laying. In the climatic chambers constant conditions were maintained (constant temperature, 70% relative humidity and a 12:12 h nyctemeral cycle which is a standard cycle in the field in Bolivia). Constant temperatures were 15, 20, 22, 25, 27, 30, 33, 35 and 37°C and for each temperature, the experiment was repeated 1 to 3 times (Table 1). Oviposition vials were hourly checked for the presence of eggs and dead mosquitoes. Individual time to oviposition and mortality were recorded throughout each entire experiment until the last act of egg-laying. Individual times to oviposition were used to estimate the model parameters. Indeed, the biological process analysed measured a combination of Beklemishev's phase 2 and some portion of phase 3 (underestimated as no searching for oviposition site was required). However, for clarity in the text, this process will still be called Beklemishev phase 2.

**Table 1:** Mean times to oviposition and proportion of ovipositing females at each constant temperature.

	Temperature								
	15°C	20°C	23°C	25°C	27°C	29°C	31°C	33°C	35°C
Number of replicates	2	1	2	2	2	3	2	2	1
Total number of mosquitoes used	100	100	200	200	180	210	200	200	100

Cohort 1	8.6	4.8	3.8	3.0	2.9	2.3	1.9	1.8	2.0
	2%	24%	18%	61%	64%	48%	52%	38%	68%
Cohort 2	9.8	5.8	4.8	4.0	3.8	3.1	2.7	2.7	2.9
	19%	34%	44%	29%	31%	48%	46%	56%	29%
Cohort 3	10.9	6.7	5.8	5.0	4.9	4.1	3.7	3.6	
	10	18%	27%	7%	4%	4%	2%	5%	
Cohort 4	11.9	7.9	6.8	6.1	5.8			4.6	4.9
	15%	14%	8%	2%	1%			1%	2%
Cohort 5	12.9	8.9	7.7						
	17%	6%	3%						
Cohort 6	13.9	10.4							
	12%	4%							
Cohort 7	14.7								
	6%								
Cohort 8	15.7								
	7%								
Cohort 9	16.8								
	7%								
Cohort 10	17.8								
	4%								
Number of ovipositing nights	10	6	5	4	4	3	3	4	4
Minimum time to oviposition	8.6	4.6	3.7	2.9	2.5	2.2	1.8	1.6	1.9
Maximum time to oviposition	18.3	10.8	7.8	6.3	5.8	4.0	3.9	4.6	4.9
GMTO (std. dev.)	12.8 (2.40)	6.4 (1.47)	5.1 (0.91)	3.5 (0.74)	3.3 (0.60)	2.7 (0.46)	2.1 (0.43)	2.4 (0.55)	2.3 (0.56)
IQR (days)	3.13	1.46	1.04	1.00	0.97	1.73	0.73	0.85	0.88

Mean times to oviposition for each cohort at constant temperatures are in days. For each temperature the table also shows, the number of replicates of experiments, the total number of mosquitoes used, the number of nights (or mosquito cohorts) from the earliest observation of laying for all mosquitoes to lay their eggs, the minimum and maximum times to oviposition recorded (in days), the general mean time (GMTO) in days to oviposition, with its standard deviation in brackets and the interquartile range (IQR) in days

Mortality data were used to draw mortality curves using the Cox model [25]. For each constant temperature, it gave the proportion of surviving mosquitoes with time. These data were used to crudely hypothesize the co-variations of the gonotrophic cycle length and mortality and their probable mutual influence on the vectorial capacity of *An. pseudopunctipennis*.

### Validation of the model

To validate the model under varying temperatures, a simulation of daily fluctuating field temperatures mimicking those in a natural resting site of *An. pseudopunctipennis *was carried out in the Binder climatic chamber with the following daily cycle: 28°C at 0 h00, and then at each following hour 29°C, 28, 27, 27, 26, 25, 25, 24, 23, 22, 22, 21, 21, 22, 22, 23, 24, 25, 25, 26, 26, ending at 27°C at 23 h00, with 50–70% RH, and a 12:12 day:night cycle beginning at 19:00 h (light time) permitting observations during working days. A new batch of mosquitoes was used and the observed time to egg-laying was then compared to the time predicted by the model.

A field validation has also been carried out in Mataral (S 18.6024, W 65.1444, altitude 1500 m), a small village characteristic of those encountered in the dry valleys of the Bolivian Andes, where human malaria occurs. A batch of field captured mosquitoes (human bait collection) was engorged on a donkey and was kept in a small natural cave in a clay cliff situated at one end of the village and where field engorged *An. pseudopunctipennis *females used to rest to mature their eggs. Temperatures where recorded using an automatic temperature recorder (Hobo RH/Temp Pro 64 k) and time to oviposition was recorded for each mosquito by regularly checking for presence of eggs. Mean observed times to oviposition were then compared to model predictions.

### Use of the model with field temperature records

To solve the model (*i.e*., equation (5) iteratively), small periods of time where temperature is considered constant are needed. In this study, two kinds of data were used. Firstly, field temperatures were directly obtained in Mataral. There, from June 2002 to January 2004, an automatic temperature recorder (Hobo RH/Temp Pro 64 k) was placed in the small natural cave (*An. pseudopunctipennis *resting place). These records enabled to use the model of gonotrophic cycle duration with precise field temperatures in input. Secondly, the model was also used to compare predictions of the gonotrophic cycle duration between four characteristic sites situated in the *An. pseudopunctipennis *distribution area, but where the only temperature records were the daily maximum and minimum records from meteorological stations. The selected sites were: (i) Sucre (S 19.063556, W 65.251031, altitude 2870 m) in the centre of Bolivia, a non endemic malaria region, (ii) Aiquile (S 18.200583, W 65.181806, altitude 2250 m) and (iii) Mataral, both in a mesothermic inter-Andean valley where *An. pseudopunctipennis *transmits malaria almost all year long, and (iv) Yacuiba (S 22.013855, W 63.678004, altitude 600 m) in the south of Bolivia, where malaria transmission is markedly seasonal. These locations differ mostly in their altitude and as such in the seasonal variations of temperature. In Aiquile and Mataral, the climate is xeric, characterized by a mean annual temperature of 18°C, with daily maximum of 39°C during the austral summer and 5°C during winter (July – August). The monthly mean temperatures are above 15°C. Rainfalls are short and violent, and occur mainly between November and March. Their annual mean is between 400 and 600 mm [26]. *An. pseudopunctipennis *is abundant, especially in the region of Mataral and malaria cases are detected all year long. In Sucre the climate is mesotropical xeric with mean annual temperature of 12.4°C and mean annual rainfalls of ≈700 mm [26]. The region is not reputed to be a malaria endemic area. The vector might be present as the altitude and climate are compatible with the vector ecology [27], but no malaria cases are registered. In fact, Sucre is considered here as good representative locality of those situated at the "cold edge" of the distribution area of *An. pseudopunctipennis*. In the region of Yacuiba the climate is tropical with mean annual rainfalls of ≈1200 mm during the austral summer. The mean annual temperature is ≈22°C [26]. The region is characterized by large temperature drops known as "surazo", a cold front from the Antarctica anticyclone ascending into the mainland of South America, generating cold humid winds [28]. These events may occur frequently in Yacuiba during the austral winter, lasting a few days each time. In Yacuiba and its surroundings, *An. pseudopunctipennis *is commonly encountered and malaria cases are numerous from November to May. Because temperatures were not always recorded with the same regularity in all localities, only computations for the three consecutive years 1998–2000 could be compared. Because hourly records of temperature were not available through the meteorological stations in these sites, they were computed as follows. Using daily temperature minima and maxima from these sites, temperatures at time *t *were computed by means of a sine wave function that take into account the daily temperature cycle and has proved to adequately mimic daily temperature oscillations in the field [29,30]. During the 24 h period of each day, the field temperature *T *can be modelled as:

(8) *T(t) = a. sin(t) + b*

where

*a *is the amplitude of the sine curve, equal to (*T*_*max *_- *T*_*min*_*)/2*,

*b *is the mean of the sin curve, equal to (*T*_*max *_+ *T*_*min*_*)/2*,

*t *the time of the day (in radians, with 1 day = 2*π*),

*T*_*min *_and *T*_*max *_are the minimum and maximum temperatures respectively, recorded in a 24 h period (or obtained from a meteorological station).

Hourly temperatures are then computed using the formula (8), replacing the time *t *by its numerical value 2*π*. *h*/24 - *π*/2, with *h *= 0,...23 corresponding to each of the 24 hours of a day. Hourly temperatures were computed like this at each of the four geographical sites under study.

### Description of gonotrophic cycle patterns with the model

**Figure 2:**
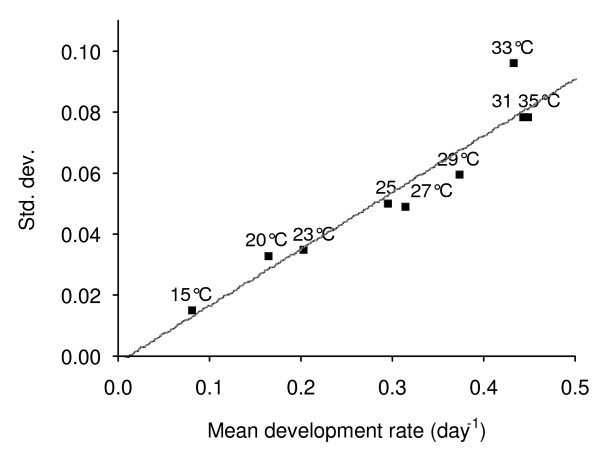
**Relation between mean development rate (MDR) and its standard deviation (SD) at various constant temperatures**. Each point is labelled by the temperature value (in °C) from the experimental chamber. The regression line is SD = -0.0017 + 0.1859 MDR, with *R*^2 ^= 0.91.

One *An. pseudopunctipennis *will lay all its eggs in a single batch, once and only during a night period (see result section). However, for a batch of mosquitoes fed at the same time, the night (post-feeding) on which all eggs are laid varies between individuals and *n *nights are needed to complete oviposition. Therefore, instead of computing a single value for the duration of the gonotrophic cycle, a more precise description would be a mathematical vector of *n *triplets of the form (*d*_*i*_, *Var(d*_*i*_*), P*_*i*_), where *d*_*i *_is the mean duration (in days) at night *i *(estimated by the model), *Var(d*_*i*_*) *is the variance of *d*_*i*_, and *P*_*i *_is the proportion of mosquitoes laying eggs at night *i*. To estimate *P*_*i *_and *n*, one can compute the overall mean development rate of the gonotrophic cycle (with its standard deviation) by mean of the "overall mean parameters" of the model (Table 2). The obtained value may then be projected in Figure 2 which represents the relationship between the mean development rate (from blood feeding to oviposition) (MDR) at one constant temperature and the corresponding standard deviation (SD) observed in the laboratory experiments. On Figure 2, each point [MDR, SD] is labelled with its corresponding experiment temperature. The regression line was SD = -0.0017 + 0.1859 MDR, with *R*^2 ^= 0.91, a high correlation coefficient. It is thus possible to infer from this relationship the "shape" (i.e., the values *n *and *P*_*i*_) of any gonotrophic cycle, from the pattern of the gonotrophic cycle on the regression line which dot is the closest to the one projected for the cycle under study. The defined pattern (*i.e*., the values *n *and *P*_*i*_) can then be read from Table 1. This suggested kind of analysis is particularly worthwhile if temperatures are low (cool season) giving a pattern of egg-laying with large variance (*i.e., n *large).

**Table 2:** Estimates of Lactin et al. model parameters.

	1^st^ mosquito cohort	2^nd^ mosquito cohort	3^rd^ mosquito cohort	4^th^ mosquito cohort	Overall mean
*ρ* (*σ*^2^)	0.01798 (0.00038)	0.01256 (0.00023)	0.00857 (0.00025)	0.0068 (0.00023)	0.01555 (0.00043)
*T*_*m*_ (*σ*^2^)	38.25 (0.0767)	38.47 (0.0927)	38.65 (0.3205)	38.78 (0.1834)	38.27 (0.1512)
Δ (*σ*^2^)	1.2007 (0.0707)	1.2006 (0.0753)	1.1471 (0.2258)	1.1211 (0.1197)	1.1182 (0.1336)
*λ* (*σ*^2^)	-1.244 (0.0167)	-1.117 (0.0082)	-1.042 (0.0067)	-1.022 (0.0054)	-1.196 (0.0164)
*T*_*upper*_	36.2	36.2	36.3	36.4	
*T*_*base*_	12.2	8.8	4.8	3.2	

Variances of estimates are in brackets. Estimates are given for each of the four first cohorts. A cohort *i *corresponds to a group of mosquitoes from the original batch that have laid their eggs on night *i*. The overall mean was computed with all data including all the mosquito cohorts (see text). *T*_*m*_, Δ, *T*_*upper *_and *T*_*base *_are in C°. *λ *is in days^-1^.

### Qualitative analysis of Beklemishev's phases 1 and 3

The model was design to predict mainly the duration of Beklemishev's phase 2 of the gonotrophic cycle. Phases 1 and 3 were not entirely taken into account and therefore the entire gonotrophic cycle duration may not always be equated to the model predictions. To size possible bias in model predictions, duration of Beklemishev's phases 1 and 3 and their variations within seasons should be estimated. Observation of development stages of mosquitoes ovaries in field captured insects may give some cues. Wild caught *An. pseudopunctipennis *were captured from human landing catches in Mataral village. Mosquitoes were caught inside and outside houses on four consecutive nights on a monthly basis from April 2002 to June 2003. These mosquitoes are part of a larger study on malaria transmission dynamics [31] and only results on ovarian development are presented here. Collected mosquitoes were identified and dissected for parity and Chistopher ovary's stage classification (stages I, II-e (e for early), II-m (m for mid), II-l (l for late), III, IV and V) [32]. Human landing catches are mosquitoes that came to a human host to feed, and as such, the developmental stage of the ovaries indicated not only the physiological age of the mosquitoes but still a rough estimate of the time spend between oviposition and refeeding (for parous females) or emergence and first feeding (for nulliparous females): The more advanced the development stage of the ovaries, the more time the mosquito has spent to come to its host.

## Results

### Modelling time to egg maturation

#### Egg-laying periods in laboratory experiments

Throughout the text, a batch of mosquitoes is referred as a group of mosquitoes blood-fed at the same time. A "cohort" represents the mosquitoes from one batch that lay their eggs at the same time (*i.e*., during the same night). Therefore, a cohort of mosquitoes is a fraction of a batch of mosquitoes.

**Figure 3:**
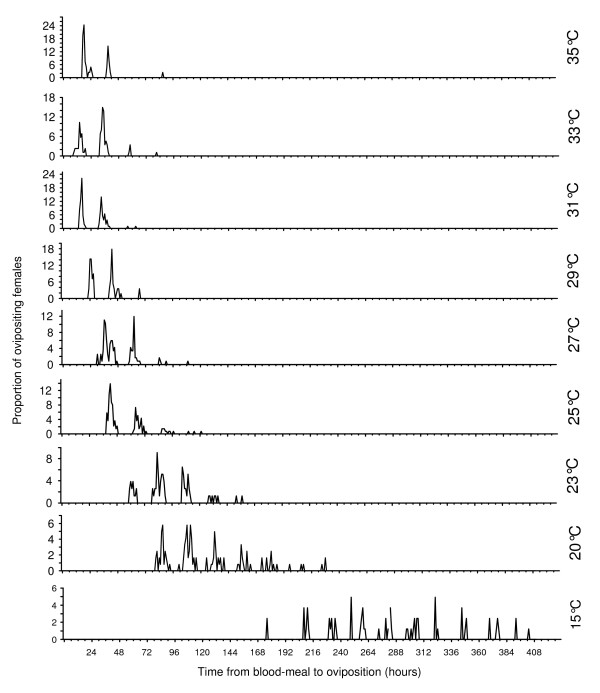
**Egg laying patterns for *An. pseudopunctipennis *at various constant temperatures**. The number of ovipositing nights is pictured by the observed "pulses" which have a pseudo Gaussian mode.

One single *An. pseudopunctipennis *laid all its eggs in a single batch, once and only during a night period, but for a batch of mosquitoes, the oviposition period lasted several consecutive nights because of individual variations in egg maturation. Therefore, the computation of a global mean time to oviposition (GMTO) is only informative because it cannot take into account the nightly pulse of egg laying. Moreover, it has a large variance, indicating a lack of precision (Table 1). Instead, at each constant temperature, a mean time to oviposition was computed for each cohort *i, i+1 *etc. These computed means, along with the percentage of mosquitoes (number of mosquitoes in the cohort/number of mosquitoes of the batch), the minimum, maximum and global mean times to oviposition are given in Table 1. The general pattern of eggs laying at various temperatures appears in Figure 3, showing the nightly pulses of egg-laying (following a pseudo Gaussian mode), the increasing times to first oviposition and increasing egg-laying durations with decreasing temperatures (and to a lesser extent with the highest temperatures).

At 15°C the rate of development was the slowest. At that temperature, the batch of mosquitoes begun to lay eggs 8.6 days (mean value) after the blood meal and the act of egg-laying lasted 10 days. In contrast, at 31°C the experiment was the shortest and lasted only three nights, and the first batch of eggs was laid as soon as 1.8 days (mean value) after the blood meal and the last one at 3.9 days. At higher temperatures (33 and 35°C), laying began 2.4 and 2.3 days (mean values) after the blood meal respectively. The duration of laying were short (four days), but a little longer than at 31°C. At 15°C the GMTO was 12.8 days while at 31°C it reduced to only 2.1 days. It remained close to this value at 33 and 35°C. The inter-quartile range which represents the time interval over which the middle 50% of the mosquitoes that have laid eggs, was greatest at 15°C (3.13 days) and reduced as temperatures until 31°C where it reached its minimum (0.73 days). At each constant temperature, the interval between two consecutive peak times was regularly 24 h, indicating a strong daily regularity in the mean timing of egg-laying.

With increasing temperatures, the night rank when a higher proportion of females laid eggs shifted from night 2–5 (at 15°C) to night 1. At 35°C, the first cohort represented 70% of the mosquitoes laying eggs and was the highest proportion in all the temperatures tested, indicating that at this temperature, the development rate reached a maximum. At 37°C, all *An. pseudopunctipennis *died before laying eggs, and the ovaries never developed beyond stage III.

### Estimation of model parameters

**Figure 4:**
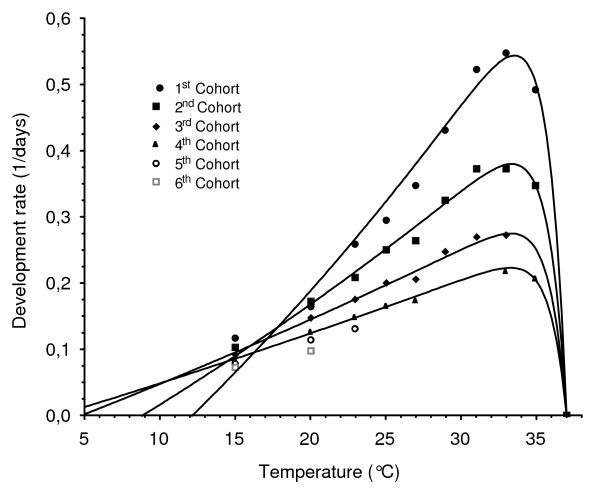
**Physiological time model for egg maturation of *An. Pseudopunctipennis***. For cohort 5, 6 and beyond, there were not enough points to estimate the Lactin *et al *function parameters.

Because the egg-laying process was not punctual in time but lasted several consecutive nights for each batch of mosquitoes, the physiological time model was adjusted for each mosquito "cohort" *i*. For the set of temperatures tested, there were sufficient data to model times of egg development for four "cohorts" (*i.e*., for all temperatures, there were sufficient data points [*r*, *T*] available for nights 1 to 4). For cohorts 5, 6 and beyond, an insufficient number of points [*r*, *T*] was available to allow the statistical estimation of the model parameters, because a number of successive ovipositing nights > 3 only succeeded with few low experimental constant temperatures. Model parameters were computed for the four first "cohorts" from [*r*, *T*] data where development rates *r *had the dimension of days^-1 ^(Table 2). The model is graphically represented in Figure 4. For all cohorts, the upper temperature *T*_*upper *_was similar, at ≈ 36.2 – 36.4°C while the base temperature *T*_*base *_ranged 12.2 – 3.2°C from the first to the fourth cohort respectively, indicating that even at very low temperatures, *An. pseudopunctipennis *may be able to develop its eggs. The value of 12.2°C may represent an "optimal" base temperature, *i.e*., the minimal threshold temperature at which an "optimal" development of eggs may begin (value for the first mosquito cohort that lay eggs).

### Validation of the model under varying temperatures

Under varying temperatures in climatic chamber, observed mean times from blood ingestion to egg-laying was 71.7 hours (std. dev. 0.9) for the first mosquito cohort, 96.2 (1.2) and 120.2 (0.9) hours for the second and third mosquito cohort respectively. On the side of the model, computed values were 75.5, 96.5 and 123.5 hours for the first, second, third mosquito cohorts respectively, indicating a remarkable similarity of the results for the two approaches.

From *An. pseudopunctipennis *maintained in cages in a field resting site situation, the observed mean time to oviposition was 96.2 hours (std. dev. 0.8) for the first mosquito cohort, 121.1 (1.3) and 144.7 (1.0) hours for the second and third mosquito cohort respectively. The model predicted 99.1, 124.2 and 147.3 hours for the first, second and third mosquito cohort respectively. Once again, observed and predicted results are very close, indicating that the model may work well to predict time to egg maturation of *An. pseudopunctipennis*.

### Use of the model with field temperature records

To simplify the interpretation, only the first "cohort" of mosquitoes was taken into account and analysed with the model. As such, results represent minima of predicted durations of the gonotrophic cycle. If values for the following cohorts are needed, the cycle duration should be increased by ≈one day (24 h) each time, in accordance with Table 1.

**Figure 5:**
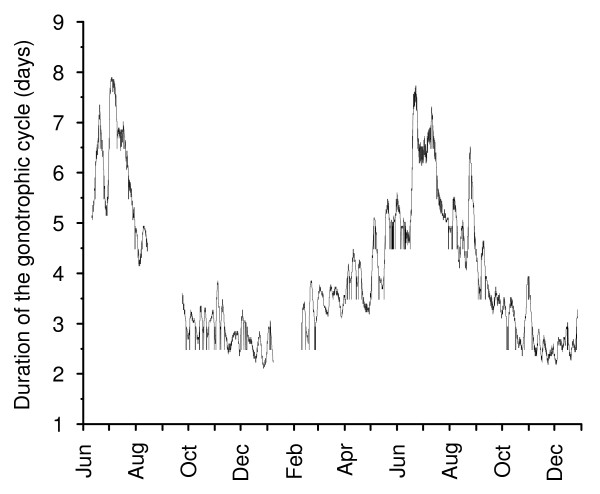
**Model predictions of length of the gonotrophic cycle (Phase 2 of Beklemishev) for *An. pseudopunctipennis *in a field resting place**. Temperature data permitting computations were collected from June 2002 to January 2004, with recording stops in September 2002 and February 2003. Cycle duration is in days.

In the cave, for the analysed period june 2002 – January 2004, the predicted duration of the gonotrophic cycle for the first "cohort" exhibited strong seasonal variations, with a predicted duration of 2.5 – 3 days during the hottest months of the year (October to February) and up to 6–7 days during the austral winter (June – August). During intermediate months the predicted gonotrophic cycle duration was 3–5 days (Figure 5).

**Figure 6:**
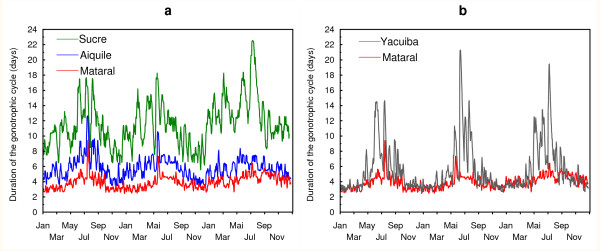
**Model predictions of length of the gonotrophic cycle (Phase 2 of Beklemishev) for *An. pseudopunctipennis *in four representative localities of Bolivia**. a. Localities of Sucre, Aiquile and Mataral, from January 1998 to December 2000. b. Localities of Yacuiba and Mataral (as a baseline comparison), from January 1998 to December 2000. Cycle durations are in days.

In the four representative geographical localities, the predicted gonotrophic cycle duration computed for the first "cohort" during the three consecutive years 1998–2000, showed the same oscillating pattern, with longest cycles during the coldest months (June to September) and shorter cycles during the hottest ones (November to March) (Figure 6). Monthly mean durations of the predicted gonotrophic cycle for these four localities are given in Table 3 and may be used as a first approach to understand cycle length durations and their variations in different characteristic regions of Bolivia. In Mataral, the predicted variations of the gonotrophic cycle duration during 1998–2000 were in accordance with those predicted in the cave during the years 2001–2002, with a minimum of ≈2.5 days on average and maximum of 5–6 days (Figure 6a). The longest monthly mean duration was predicted in July (5 days), while the shortest was predicted during the hot season from November to March (3.3 – 3.6 days) (Table 3). The associated variances were always small (< 1) indicating stability in the monthly cycle durations. In Sucre, where temperature conditions are the coldest of the four studied sites all year long, the minimum predicted cycle duration was ≈8 days on average, and the maximum ≈12–14 days (with extreme at 18 – 24 days) (Figure 6a). Mean monthly predicted durations were always > ≈ 10 days. Even when variances are large, indicating that cycle duration may sometimes be shorter, adequate temperature conditions did not last long enough to allow stability and thus efficient malaria transmission. In Aiquile, which is a small town close to Mataral, but at a higher altitude, the predicted variations exhibited the same seasonal pattern, but were more pronounced. Minimum and maximum predicted values for cycle length oscillated between an average of 4–5 and 7–8 days respectively (Figure 6a). In Yacuiba, (in the extreme south of Bolivia), predicted cycle durations were identical to those predicted in Mataral during the summer, with 2.5–3 days on average. However, they were quite different during winter seasons, with higher predicted cycle durations in Yacuiba (≈8 days on average, with extreme values up to 14 – 22 days) (Figure 6b). The increase in predicted cycle duration is due to "surazo" events that cause important drop in temperature. Therefore, with predicted monthly mean cycle durations > 10 days in June-July and > 6–7 days during the preceding and following months, with large variances (Table 3), malaria transmission is unlikely during the winter season in such a region.

**Table 3:** Duration of gonotrophic cycle of *An. *

	Sucre	Aiquile	Mataral	Yacuiba
January	10.3 (5.28)	4.9 (0.42)	3.3 (0.31)	3.4 (0.08)
February	11.9 (2.67)	5.7 (0.69)	3.5 (0.37)	3.6 (0.27)
March	11.9 (8.03)	5.3 (0.77)	3.3 (0.15)	3.7 (0.45)
April	12.3 (2.94)	5.9 (1.00)	3.7 (0.22)	4.8 (1.58)
May	12.6 (10.45)	6.8 (1.63)	4.4 (0.68)	7.4 (7.48)
June	14.0 (3.64)	7.0 (0.65)	4.7 (0.17)	10.9 (18.71)
July	15.2 (16.86)	7.5 (2.80)	5.0 (1.07)	10.1 (14.32)
August	12.5 (16.90)	6.8 (2.78)	4.3 (1.06)	6.3 (14.34)
September	13.3 (3.09)	6.0 (0.58)	4.4 (0.59)	6.6 (1.34)
October	9.2 (2.13)	5.0 (0.64)	3.8 (0.61)	4.2 (0.46)
November	8.9 (3.30)	4.6 (0.73)	3.6 (0.45)	3.8 (0.40)
December	9.8 (4.25)	4.6 (0.45)	3.5 (0.39)	3.3 (0.18

*pseudopunctipennis *in four representative localities of Bolivia, predicted by the model. Values are monthly means (in days) and variances (in brackets) for the first cohort of mosquitoes and as such represent minimum values of the gonotrophic cycle duration. *Sucre *is a locality situated in altitude with marked seasons and cool summer; *Mataral *is a characteristic locality of the mesothermic Andean valleys of Bolivia where malaria transmission is active; *Aiquile *is a similar locality but cooler and *Yacuiba *is a characteristic locality where winter "surazo" events act.

### Mosquito mortality

**Figure 7:**
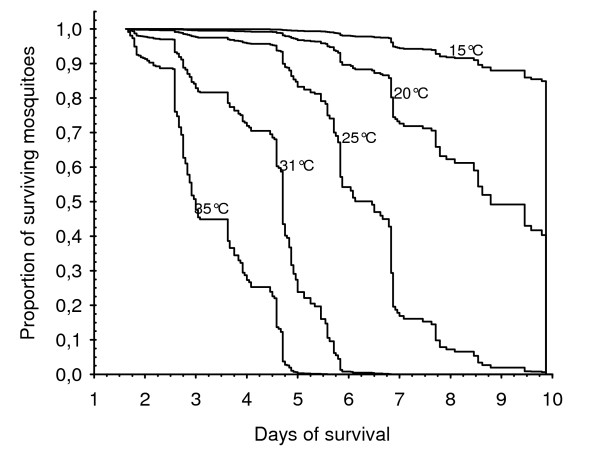
**Cox model representation of *An. pseudopunctipennis *mortalities at various constant temperatures**. The model expresses the instantaneous risk of mortality according to the temperature.

Mortality patterns from the Cox model at various constant temperatures are presented in Figure 7. At 15°C, mortality was low and 90% of the mosquitoes survived > 10 days. Inversely, at 35°C, all mosquitoes died within five days. There was a constant increase of the mortality rate with increasing temperature. Fifty percent of the mosquitoes (*P*_50_) were dead at day 3 at 35°C, day 4.5 at 31°C, day 6.5 at 25°C and day 9 at 20°C. There was a very good linear relationship between the *P*_50 _and temperatures (*R*^2 ^= 0.99).

### Qualitative analysis of Beklemishev's phases 1 and 3

Monthly captures of females and ovarian development stages at the time of capture are presented in Table 4 for nulliparous females and Table 5 for others. Taking into account significant data (*i.e*., months with at least > 100 dissected females), there was an increase in the proportion of nulliparous females with ovaries more developed (stage II-m and II-l) during the coldest months (July and August), as compared with those less developed (i.e., at stage I or II-e). More females at stage 1 were captured coming to host during the hot season. This tendency to "older ages" at biting during the coldest months was also noticed in the group of the parous females for which stages II-e are less numerous when compared with stages II-m during the cold season. It is obvious that ovaries develop slower at lower temperatures, but this does not imply that mosquitoes should also delay their moment of biting. In fact, mosquitoes seem to take more time to come to host when temperatures are colder. This phenomenon could be attributed to a fall in mosquito activity when temperatures are cold, which means that seeking a host takes more time. During the host-seeking period, ovaries develop according to temperature. If ovaries are more developed at biting time during the cold season, and if the mosquitoes take even more time to seek their hosts, it means that the time elapsed from oviposition to biting might be significant during such a season. A non-negligible proportion of females with ovaries at stages III, IV and even V were captured (the sum of these females ranged 5 – 15% of the total captured), and ≈10% of the captured females were at stage II-l, indicating that (i) some *An. pseudopunctipennis *females needed more than a single blood-meal to mature its eggs and (ii) the time between egg-laying and re-feeding could be appreciable. As such, phase 1 of Beklemishev may be of significant duration for parous and nulliparous *An. pseudopunctipennis *and model predictions should be adjusted upward.

**Table 4:** Nulliparous *An. pseudopunctipennis *females in each Christopher's stage category.

	Total captured	% in Stage 1	% in Early stage 2	% in Mid stage 2	% in Late stage 2
April 2002	176	15.3	45.5	39.2	0.6
May	283	21.6	50.9	27.6	0
June	684	29.4	47.2	22.1	1.3
July	401	12.0	37.7	47.6	2.7
August	279	4.7	17.6	72.8	5.0
October	495	24.2	50.5	25.3	0
November	13	7.7	61.5	30.8	0
December	26	28.5	34.6	26.9	0
January 2003	57	38.6	52.6	8.8	0
February	20	5.0	45.0	50.0	0
March	9	22.2	33.3	44.4	0
April	42	21.4	57.1	21.4	0
May	399	19.0	46.9	34.1	0
June	356	15.4	61.5	22.8	0.3

Total	3240	19.9	45.9	33.1	1.1

Mosquito females were captured in Mataral by human landing catches from April 2002 to June 2003. Table shows monthly total catches and monthly proportion of nulliparous females in each stage.

**Table 5:** *An. pseudopunctipennis *in each Christopher's stage II to V.

	Total captured	% in Early II	% in Mid stage II	% in Late stage II	% in Stage III	% in Stage IV	% in Stage V
April 2002	463	27.0	52.1	13.8	5.0	1.5	0.6
May	287	41.8	51.6	3.1	1.0	2.4	0
June	964	39.2	47.3	10.2	1.2	2.1	0
July	321	8.1	69.8	6.9	8.7	4.0	2.5
August	280	2.9	73.9	11.1	7.9	2.9	1.4
October	642	40.5	38.0	10.6	6.1	2.2	2.6
November	52	25.0	53.8	11.5	0	9.6	0
December	29	27.6	69.0	3.4	0	0	0
January 2003	168	72.6	14.3	5.4	0.6	6.0	1.2
February	39	23.1	56.4	12.8	0	7.7	0
March	82	61.0	37.8	1.2	0	0	0
April	301	54.5	38.2	3.7	0.7	1.3	1.7
May	730	20.3	58.9	10.5	4.5	5.1	0.7
June	388	45.6	43.6	7.0	1.3	2.3	0.3

Total	4396	36.6	53.7	9.8	3.8	3.1	1.0

Mosquito females (individuals from stage II [except nulliparous females] to stage V) captured in Mataral by human landing catches from April 2002 to June 2003. Table shows monthly total catches and monthly proportion of mosquito females in each stage

## Discussion

For *An. pseudopunctipennis*, mean observed values of the duration of the gonotrophic cycle at constant temperatures in laboratory conditions are in agreement with results from *An. albimanus*, another Neotropical *Anopheles *[33]. Egg maturation in *An. pseudopunctipennis *is temperature dependant and the egg-laying is light dependant: These night active mosquitoes stop laying eggs during day time. One single *An. pseudopunctipennis *will lay eggs once. This behaviour is not shared by all mosquito species. For example, one *Ae. aegypti *placed in the same experimental conditions as for *An. pseudopunctipennis *will lay eggs for two or three consecutive days and may even hold its eggs ([34], Lardeux, pers. obs.) complicating even more the computation of the duration of its gonotrophic cycle. As for a batch of *An. pseudopunctipennis *is concerned, several consecutive night are needed until the last mosquito has laid its eggs. The range of nights over which oviposition takes place is wider when temperature decreases, with a minimum variation of three nights at 31°C which seems to be the temperature at which egg maturation is the shortest (range 1.8 – 3.9 days). In general terms, the variance is larger with decreasing temperatures. So during the cold season, not only is the mean time for the first cohort of mosquitoes to lay eggs late, but also few mosquitoes will lay eggs each consecutive night.

A model of physiological time development should take into account this number of successive ovipositing nights. To achieve this, the present model contained several sub-models corresponding to the successive mosquito cohorts. However, another way of using the model could be to parameterize it with the overall mean estimates of the parameters of Table 2 and simply weight it by the proportion of mosquitoes that laid eggs in the different cohorts. Four cohorts were (sub-) modelled, representing the egg development history of ≈80–100% of mosquitoes when temperatures are > 20°C. When temperatures are low (< 20°C), *An. pseudopunctipennis *is basically inactive and transmission greatly reduced, even disappearing. As a consequence, the cohorts observed in the laboratory at low temperature are almost virtual in nature. Therefore, the four cohorts which are part of the model are sufficient to describe the gonotrophic cycle duration in the temperature range where *An. pseudopunctipennis *usually lives, and they may account for almost all mosquitoes laying eggs.

For simplicity of the analysis, only results from the first cohort of *An. pseudopunctipennis *were presented, giving predicted minimum values for the gonotrophic length. However, it is easy to complete the analysis for that mosquito as each following cohort appears 24 h later.

The minimum number of cohorts was three and was observed at optimal (high) temperatures, and is therefore a minimum number to correctly describe the dynamics of the gonotrophic cycle of *An. pseudopunctipennis*. As such, the gonotrophic cycle duration cannot be summarized by only one single value (*i.e*., an overall mean of all cycle lengths of all mosquitoes from all the cohorts) as it is usually done. The suggested presentation of the gonotrophic cycle duration by means of a mathematical vector of *n *triplets (*d*_*i*_, *Var(d*_*i*_*), P*_*i*_), representing each of the *n *cohorts laying eggs with their relative proportion *P*_*i *_and the mean value (and variance) of the cohort cycle duration *d*_*i*_, (*Var(d*_*i*_*)) *may be an alternative. However, such a vector should be computed for each period of varying temperature, at least on a season basis or less (for example, on a monthly basis) if temperature variations are on a shorter scale. These findings are important in precise modelling of transmission and should be taken into account whatever the mosquito species under study. Some attempts have been made to incorporate the influence of temperature via the gonotrophic cycle in transmission dynamics modelling [35]. These kinds of models could be improved using a physiological time approach and, for the gonotrophic cycle duration, the use of the suggested mathematical vector.

In the field, the situation may be even more complicated as a significant proportion of *An. pseudopunctipennis *(≈5%) has tendency to take several blood meals to complete the maturation of its eggs, thus increasing the overall vectorial capacity [31]. If so, the risk of transmission is not only directly linked to the gonotrophic cycle duration. At least some mosquitoes may transmit more frequently than predicted. Transmission may thus occur and this may partly explain the (low) levels of transmission by *An. pseudopunctipennis *despite apparently weak components of its vectorial capacity [31].

The time spent to search for a host (phase 1 of Belekmishev) may also be significant for *An. pseudopunctipennis *as indicated by the significant proportion of mosquitoes with "advanced" ovary development at the time of (re)feeding. If so, predictions from the physiological time model are minimum values of the cycle duration for *An. pseudopunctipennis *and they should be slightly adjusted upwards to give more accurate estimations. This is particularly true during winter conditions when temperatures are lower. The model also gives a value of ≈36°C for the upper temperature (*i.e*., temperature at which rates are the fastest). This value is biologically realistic and in accordance with the temperature of 35°C of the experiments where apparently the development rate was the fastest for at least 70% of the tested mosquitoes. As such, the computed value indicates that the model may correctly describe the phenomenon.

Indeed, other factors than the gonotrophic cycle length have an impact on the vectorial capacity, such as mosquito densities, mosquito longevity, and the duration of the extrinsic cycle of the parasite. All of them are more or less influenced by temperatures [36-38]. The definition of vectorial capacity assumes that the length of the gonotrophic cycle and survival are independent parameters. It is not always the case and present results on *An. pseudopunctipennis*, although in laboratory conditions, indicate that they are not: as temperature decreased, the length of the gonotrophic cycle and the mosquito survival increased. If *An. pseudopunctipennis *longevity is higher when temperatures are low, it may then live long enough to transmit, even if it takes longer to complete its gonotrophic cycle. The extension in survival at low temperatures could in theory nullify the decreasing effect of a long gonotrophic cycle on transmission. As vectorial capacity (and *R*_0_) is more influenced by survival than by the gonotrophic cycle length, it is probable that effects of low temperatures on the gonotrophic cycle will be compensated (and even overwhelmed) by increased survival, generating transmission. At higher temperatures, higher vector mortality may limit the transmission although the gonotrophic cycle is shorter. The question is then if the mosquitoes have enough time to transmit before dying. In nature, other ecological factors than temperature may interfere with mortality, complicating such analysis. Therefore, transmission variations cannot be simply interpreted by only variations in the gonotrophic cycle length. However, for *An. pseudopucntipennis*, model predictions of the gonotrophic cycle duration are qualitatively correlated with malaria transmission patterns observed in the four sites studied. Therefore, the model can in a rough and cautiously first approach be used to better understand transmission variations in the distribution range of the vector in Bolivia. However, more research is needed to completely model the influence of temperature on *An. pseudopunctipennis *survival and to include the results in a temperature-dependant model of vectorial capacity embodying not only survival, but also the parasite extrinsic cycle and the gonotrophic cycle as others temperature-dependent phenomenon. It is likely that temperature will be revealed to be an important factor that will explain, along with a poor human biting index [31], why *An. pseudopunctipennis *is a poor malaria vector as compared to other *Anopheles *species (*An. darlingi *in the Amazon region of Bolivia for example, or other African species).

Temperature might also account for the unstable time/season pattern (in both the long and short terms) of *An. pseudopunctipennis *transmission in reputed malaria areas. Used with field temperature records, the model showed that the predicted gonotrophic cycle of *An. pseudopunctipennis *in Bolivia is variable following a seasonal pattern and that the variations of amplitudes depend also on the geographic location. The predicted variations are more pronounced at high altitudes where temperature conditions may vary greatly, and also in some lowland places where climatic phenomenon such as the "surazo" prevails (*i.e*., in the foothills of the Andes, from the south of Bolivia up to the latitude of Santa Cruz (≈ 17.8°W). The "surazo" events have a strong influence in the Bolivian lowlands on the distribution of biocenosis and ecosystems and are responsible for the southern limit of most Amazonian animals and plants in the lowlands [26]. Moreover, these polar air masses and cold winds can lower the ambient temperature by more than 20°C in one day. In winter, the frequency of such events is more or less of twice per month and each lasts a mean of 2–6 days [39]. The locality of Yacuiba is characteristic of "surazo" events. There, the gonotrophic cycle of *An. pseudopunctipennis *can be very long, as illustrated by the model predictions, due to these periods of cold air influence, limiting transmission strength. On the other hand, during summer, cycle duration can be very short, improving the vectorial capacity of the mosquito. As a consequence, malaria transmission is indeed more active during summer as confirmed by epidemiological data of human cases [40]. "Surazos" are less active in the inter-Andean valleys. As such, regions where the gonotrophic cycle of *An. pseudopunctipennis *is more or less constant and short are the mesothermic Andean valleys for which Mataral and Aiquile could be prototype localities. Results of ecological niche modelling for *An. pseudopunctipennis *using MAXENT algorithm [41] showed that this mosquito vector is well present in these ecological regions, where temperature conditions are also optimal for a short gonotrophic cycle. Epidemiological data confirm that these valleys account for the majority of malaria cases due to *An. pseudopunctipennis *[40]. With predicted short cycle durations and small variances all year long, transmission in Mataral is likely to be almost constant if parasites and mosquitoes are present in sufficient densities. In Aiquile, malaria transmission is more likely during summer but less active than in Mataral. At the edges of its distribution range, *An. pseudopunctipennis *encounters less favourable climatic condition for a short gonotrophic cycle and as a consequence, epidemiological data confirm that transmission is less active in these areas. In Sucre for example, the predicted gonotrophic cycle length is always > 10 days, impeding malaria transmission (even if mosquito survival may be increased by low temperatures, the extrinsic cycle of the parasite is too long to enable transmission). A brief outline of the various geographical and seasonal situations encountered in Bolivia and their impact on the predicted gonotrophic cycle duration of *An. pseudopunctipennis *is summarized in Table 3 which could be used as a first rough approach for cycle duration estimates throughout Bolivia.

It is known that insects avoid extreme temperatures and may respond to such stress with a kind of behavioural regulation. Mosquitoes can regulate their body temperature by moving into adequate areas [42]. Within the normal range of temperature in which they are active, mosquitoes have a preferred range in which, given the choice, they tend to remain for relatively long periods, in particular when they digest their bloodmeals. As such, one might doubt the model predictions if inadequate temperature records are used to run it. However, with the Mataral example, the model gave similar predictions when using hourly temperatures records in a field resting site (the cave) for *An. pseudopunctipennis *to when using the maximum/minimum values from a close meteorological station. Consequently, meteorological station records can reasonably be used to model the gonotrophic cycle duration in many field situations in Bolivia.

The model uses a S-shaped curve for developmental rates which embodies the assumption that developmental rates adjust instantaneously to changes in temperature. This is reasonable given the gradual nature of field temperature curves. Another assumption is the lack of synergism due to fluctuating versus constant temperatures. This second assumption is more tenuous [43] but has been found to be approximately true for many situations, and in particular for *An. pseudopunctipennis *for which model predictions in variable temperature experiments were identical to the observed values for the cycle duration.

Long before, other attempts have been made to model the duration of the gonotrophic cycle duration of mosquitoes taking into account temperature variations with empirical functions [37,44]. The flexibility of the present model can encompass complex situations of variable temperatures and can be adapted to a variety of bloodsucking insects. Climatic conditions and amongst them temperature variations are more and more taken into account in the light of the global warming phenomenon to predict changes in species niche occupation and also to model changes in vector transmission risks [45-48], even in Europe [49,50]. The model presented here is a first step toward the modelling of malaria risk transmission in Bolivia that could be used in a GIS analysis to map the vectorial capacity of *Anopheles pseudopunctipennis *(or better, the basic reproduction index *R*_0 _of the transmitted *Plasmodium *parasite that encompasses all the parameters of the vectorial capacity of the mosquito and some disease characteristics) [38].

## Conclusion

The present study gives a general approach to the computation of the gonotrophic cycle duration of *An. pseudopunctipennis*. This approach, based on a physiological time model, is also a framework that can be used with other bloodsucking insects. The model presented may be run jointly with the usual field approaches to refine the predictions. The cycle duration cannot be given as one single value (except with a high variance), but is better represented by a series of values based on the daily percentage of mosquitoes from one "feeding batch" that might lay eggs. In Bolivia, model predictions and the sources of variations encountered within the model (time, season, location, variances of parameters) partly explain the observed variations in malaria transmission (and its unstable behaviour). However, a more complete and refined approach to explore variation in malaria transmission would take into account temperature effects on the other components of the vectorial capacity in particular on the mosquito survival and on the extrinsic cycle of the parasite.

## Competing interests

The authors declare that they have no competing interests.

## Authors' contributions

FL designed the study, did the field work, participated in the laboratory experiments, analysed the data and drafted the manuscript. RT carried out the laboratory experiments on *An. pseudopunctipennis *cycle duration and helped in capturing wild *An. pseudopunctipennis *in Mataral. VQ and TC participated in the laboratory experiments and in collecting wild mosquitoes in the field. TC participated in designing the study and supervised the field experiments. All authors read and approved the final manuscript.
